# The ameliorative effects of *Alpinia officinarum* rhizome hydroalcoholic extract on cisplatin-induced testicular toxicity in rats

**DOI:** 10.5935/1518-0557.20220017

**Published:** 2023

**Authors:** Atefeh Ashtari, Firoozeh Niazvand, Narges Chamkouri, Asma Mohammadi, Alireza Balizadeh Karami

**Affiliations:** 1 Cellular and Molecular Research Center, Medical Basic Sciences Research Institute, Ahvaz Jundishapur University of Medical Sciences, Ahvaz, Iran; 2 Department of Anatomical Sciences, Faculty of Medicine, Ahvaz Jundishapur University of Medical Sciences, Ahvaz, Iran; 3 Assistant Professor, Department of Anatomical Sciences, Abadan University of Medical Sciences, Abadan, Iran; 4 Assistant Professor, Department of Biochemistry, Abadan University of Medical Sciences, Abadan, Iran; 5 Comprehensive Research Laboratory of Abadan University of Medical Sciences and Health Services, Abadan, Iran; 6 Student Research Committee, Abadan University of Medical Sciences, Abadan, Iran

**Keywords:** *Alpinia officinarum* rhizome extract, chemotherapy, cisplatin, testis damage

## Abstract

**Objective:**

In this study we evaluated the influence of *Alpinia officinarum* rhizome extract (AO) on the alleviation of testicular damage induced by cisplatin in rats.

**Methods:**

The study groups included the control group, AO-administered group, cisplatin-administered group, and three groups administered with cisplatin and AO (different concentrations of 100, 200, and 400 mg/kg). On the 14^th^ day we removed the testes of the rats, and the testicular organ parameters were measured. Moreover, through the malondialdehyde concentration we assessed the oxidative stress and superoxide dismutase (SOD) activity of the testes and ran a histopathological analysis.

**Results:**

The results demonstrated that cisplatin-induced oxidative stress and severe testicular damage on the AO-administered group showed no harm compared with the control group. AO- treatment in cisplatin-received rats led to the reduction of oxidative stress, enhancement of SOD activity, and prevention of testicular damage. The lowest testis damage was attributed to the group which received 400 mg/kg of AO compared to 100 and 200 mg/kg.

**Conclusions:**

Overall, the Cis+/AO+400 group had the best antioxidant effect. The findings could lead to changes in cancer care guidelines that incorporate phytochemicals, making cancer therapies safer.

## INTRODUCTION

We know that cancer is one of the most important worldwide health issues (Azadani *et al*., 2021; Faraji Dizaji *et al*., 2020; Nouri *et al*., 2021). Numerous methods and drugs have been developed to fight it (Abasian *et al*., 2019; Asadi *et al*., 2019; Bazli *et al*., 2017; 2020; Wong Jang *et al*., 2020; Radmansouri *et al*., 2018; Rouhani *et al*., 2018; Sharifianjazi *et al*., 2020). Cisplatin (Cis) is among the most commonly used anticancer drugs for solid tumors and hematological malignancies, including cancer of the head and neck, lungs, testicles, ovaries, and bladder. Cis can interfere with normal cell cycles and cause *in vivo* oxidative damage (Ghaferi *et al*., 2020; Zahednezhad *et al*., 2020). In the past few years, research has demonstrated that the use of Cis in clinical settings has been restricted by several serious side effects, such as severe kidney damage, gastrointestinal toxicity, and ototoxicity (Fetoni & Astolfi, 2020; Lu *et al*., 2020; Yilmaz *et al*., 2022). Male reproductive toxicity, specifically testicular injury, is also reported (Tian *et al*., 2018).

Male hormones secreted by the testes have a significant function in the growth and maturation of male reproductive organs and male sexual characteristics (Holtof *et al*., 2021). Aksu *et al*. (2016) found that Cis could cause irregular alterations in sperm motility and density, oxidative stress, degeneration and apoptosis of testicles. The study by Fouad *et al*. (2017) showed that 10 mg/kg Cis dramatically decreased serum levels of testosterone in rats. Furthermore, Cis radically raised testicular tissue oxidative stress, contributing to testosterone synthesis disorder (Iman *et al*., 2017). Along with abnormal changes in testis/sperm parameters, Cis can have adverse effects on the level of follicle-stimulating hormone (FSH), luteinizing hormone (LH), and testosterone levels in rat serum (Fallahzadeh *et al*., 2017).

As an essential sexual androgen, testosterone has a vital activity in reproducing and preserving vital organs’ functionality. Testosterone is produced in the Leydig cells, which are a source of several testosterone biosynthetic enzymes (Xia *et al*., 2020). Cis can cause dysfunction of the Leydig cells and disorders of testicular steroidogenic synthesis in animals, impeding testosterone production, thus causing infertility (Tian *et al*., 2018). Aksu *et al*. (2016) reported that Cis could cause oxidative stress in testicular tissues. The imbalance between the oxidative and antioxidative potential in tissues is represented by oxidative stress. Mitochondria consistently form reactive oxygen species (ROS) under physiological conditions, including superoxide anion (O_2_), and hydrogen peroxide (H_2_O_2_), hydroxyl radicals (•OH) (Yin *et al*., 2017). Zhao *et al*. (2014) stated that Cis could decrease the weight of epididymal and testis, sperm count and motility, as well as glutathione peroxidase (GSH-Px) and SOD activity, also raising the amount of malondialdehyde (MDA) in rat testis.

The *Alpinia officinarum* (AO) plant species belongs to the Zingiberaceae family, and it is a perennial herb with antibacterial, spasmolytic, and antiphlogistic properties, primarily known in Asia (China, India, Indonesia, Japan, Malaysia, and Thailand), as a medicine produced from rhizomes and roots, and is used as a tincture or tea (Yoo *et al*., 2021). The phyto-pharmacological efficiency of A. galanga was well evaluated as having antioxidant and anticancer effects (Chouni & Paul, 2018; El-Hadidy *et al*., 2020). Antioxidants support DNA and essential molecules from oxidative damage and can enhance the efficiency of sperm, thus increasing male fertility (Heidari *et al*., 2021). Hence, biochemical and nutritional factors are significant in treating reproduction and sub-fertility disorders (Mazaheri *et al*., 2014).

The hypothesis of this study is that AO can inhibit Cis-induced damages to testes by the improvement of metabolism as well as aiding in antioxidant defense mechanisms resulting from its antioxidant potential. With this regard, Cis-induced testis injuries were evaluated using an intraperitoneal injection of Cis, while the administration of AO was intragastric.

To test the above hypothesis, at first, we examined the changing of body weight and testis weight of the groups and also, we observed changes of testicular pathology and detected oxidative stress-related indicators including MDA and SOD in rat testes. Furthermore, we measured the level of biochemical hormonal marker including testosterone, LH, and FSH in the serum samples of the rats.

## MATERIALS AND METHODS

### Preparation of AO

AO was purchased in December 2020 from the local market in Bushehr, Iran. To eliminate any contaminants, the plant was washed twice with distilled water and air-dried at room temperature. The newly dried rhizomes were then ground in order to produce a powder. Dried rhizomes were blended with ethanol (95% v/v) to prepare the hydro-alcoholic extract. A Whatman paper filter (grade 4) was used to filter the blend. Utilizing a vacuum rotary evaporator at 50°C for 10 min, the hydro-alcoholic extract was condensed (Aksu *et al*., 2016; Fouad *et al*., 2017). Varying concentrations of 100, 200, and 400mg/kg were provided for AO. Before laboratory testing, the extract was kept in a refrigerator at 4°C (Lee *et al*., 2009; Mazaheri *et al*., 2014).

For phytochemical analysis of the AO extraction, we studied the concentration of total phenol and total flavonoid. To determine the concentration of total phenol according to Wolfe *et al*. (2003), 0.25mL of the extract was added to 1 mL of deionized water and mixed to 0.25mL Folin-Ciocalteu, and then the concentration of total phenol was measured using the UV-Vis spectrophotometry at a wavelength of 760nm. To measure the flavonoid concentration, 1mL of extract sample was added and mixed with 1 mL of aluminum chloride (AlCl_3_), and the concentration of flavonoid was determined by UV-Vis spectrophotometry at a wavelength of 510nm (Luximon-Ramma *et al*., 2002). Total flavonoid concentration and phenolic concentration as the phytochemical analysis were 13.92±0.07mg and 34.18±0.25mg, respectively.

### Animal model

48 male Wistar albino rats with the weight of 250 to 300g were obtained from the animal housing of Ahvaz Jundishapur University of Medical Sciences, Abadan Medical Science. The rats were held for one week in a standard laboratory setting with a thermoregulated temperature between 20 to 25°C and automatic illumination of 12h light/12h darkness. Throughout the procedure, they were supplied with adequate nutrition and proper ventilation and had unlimited access to water and food. The Institutional Animal Care and Ethics Committee of Ahvaz Jundishapur University of Medical Sciences certified all the testing methods (Ahvaz, Iran).

### Experimental design and treatment

**Figure 1:**
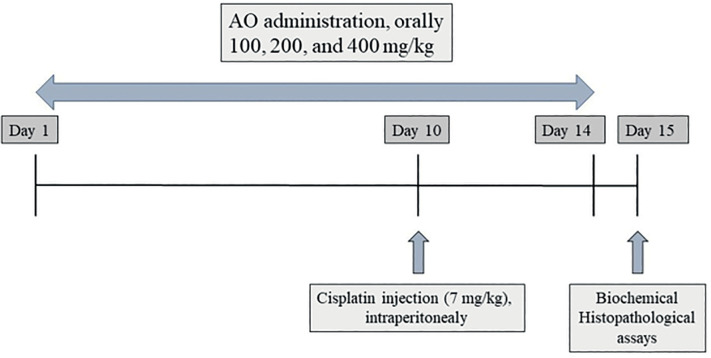
Study design diagram for protective effect of *Alpinia officinarum* (AO) on cisplatin-induced testicular toxicity.

The rats were divided into five groups (n=8) by a random categorization. In Group I (control), the rats were administered normal saline by gavage for 14 days and received a single intraperitoneal Cis injection (7 mg/kg) after 10 days. Group II (Cis^-^/AO^+^400) consisted of rats who underwent a daily oral regime of only hydroalcoholic extract of AO with a concentration of 400 mg/kg for 14 days with no Cis injection. The rats in Group III (Cis^+^/AO^-^) were administered a single Cis (7 mg/kg) intraperitoneal injection on day 10. The rats in Group IV (Cis^+^/AO^+^100), Group V (Cis^+^/AO^+^200), and Group VI (Cis^+^/AO^+^400) received Cis (7mg/kg) on the 10th day and AO for 14 days in the concentrations of 100, 200, and 400mg/kg, respectively. AO was delivered one hour prior to the Cis treatment in the groups that received Cis and AO. Various tests were performed on the 14^th^ day. The entry and exit requirements were male rats without any treatment before starting the experiment and using healthy rats without anatomic anomalies (Figure 1).

### Sample collection

The rats were put in diethyl ether distributed static inhalation cabinets. After a few minutes, when the rats were breathing slowly, the rats were brought out and set on the console. Then, by exsanguination we collected blood samples. Thereafter, the rats were slaughtered, and the testicles were rapidly removed for weighing and testicular organ parameter measurement (testicular wet weight/body weight). Out of eight rats in each group, we obtained bilateral testes, for protein extraction and detection of biochemical markers, the right testes were maintained at-20°C (Tian *et al*., 2018).

### SOD and MDA Assessment

We then placed one gram of testicular tissue specimen in a 4-mL buffer solution (1/5). The testes were homogenized to acquire a ratio of 1:10 (wt/vol) of the complete homogenate in the Teflon-glass homogenizer with a buffer comprising 1.5 percent potassium chloride, to assess lipid peroxidation and antioxidant enzymes function on the testicular tissue. Using 13,000g centrifugation for 1 hour, the homogenate was processed for evaluation. The procedure defined by Ohkawa *et al*. (1979) was employed for the assessment of MDA amounts. Tissue SOD activity analyses were carried out in compliance with the procedure indicated by Sun *et al*. (1997).

### Biochemical analysis

A sensitive rat kit (Cusabio Biotech Co. LTD) was used to assess FSH and LH, employing a double antibody enzyme-linked immunosorbent assay (ELISA). Serum testosterone was assessed using standardized laboratory techniques (Mino bine human kit, USA).

### Histopathological analysis

We used 10% (w/v) buffer formalin to fix the testis specimens during four days, followed by processing through alcohol series at the concentrations of 70%, 90%, and 100%. Thereafter, the samples were embedded in wax by a Leica Automated Tissue Processor device (TP1050, Germany). Each block was sliced into sections with a thickness of 5 µm; then we used xylene to dewax the sections. A descending alcohol series were employed for rehydration. Staining of the prepared slides was performed by Harris hematoxylin and eosin (H&E) (Soliman *et al*., 2014). We analyzed the tissue structure under a light microscope (Olympus, U-MDOB, Japan).

### Statistical analysis

For analytical data processing we used the SPSS 19 (Chicago, USA) software. The used the mean value±SD of the measured data. We used the one-way ANOVA accompanied by the post-hoc Tukey test to compare the groups, and the statistical significance was considered to be *p*<0.05.

## RESULTS

### General toxicity evaluation

**Figure 2:**
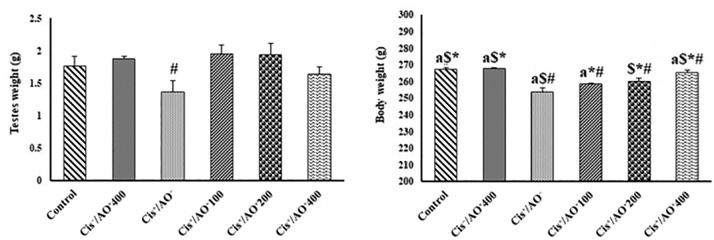
Body weight changes and testis weight changes in different groups

Figure 2 depicts the changes in body weight and testis weight. Body weight loss was obvious after Cis administration began, and AO had no effect on it. According to the findings, the Cis^-^/AO^+^400 group had no meaningful changes in body weight when compared to the control group; while the Cis^+^/AO^-^ group had a significant reduction in body weight when compared to the control group. Body weight in the Cis^+^/AO^+^100, Cis^+^/AO^+^200, and Cis^+^/AO^+^400 groups showed a reduction when compared to the control group. The amount of body weight loss in these groups was lower compared to the group treated with Cis^+^/AO^-^. The testis weight in the Cis^-^/AO^+^400 group exhibited an increase in comparison with the control group. The Cis^+^/AO^-^ group showed a considerable reduction in testis weight in comparison to the control group. Testis weight in the Cis^+^/AO^+^100 and Cis^+^/AO^+^200 groups showed a remarkable increase in comparison with the control group, while the Cis^+^/AO^+^400 group exhibited a reduction.

#: *p*<0.05 *vs*. control group,

*: *p*<0.05 *vs*. Cis^+^/AO

$: *p*<0.05 *vs*. Cis^+^/AO+100

a: *p*<0.05 *vs*. Cis^+^/AO+200.

### Testes biochemical hormonal marker

**Figure 3:**
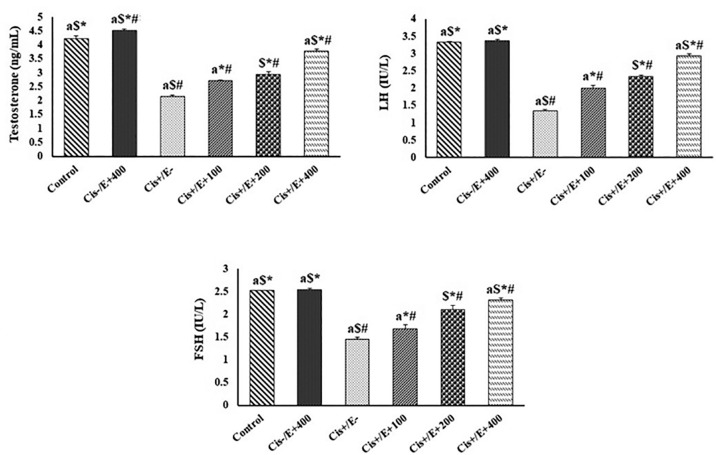
The levels of the biochemical hormonal marker for testes damage in different studied group

Figure 3 depicts, the levels of biochemical hormonal marker for testes injury, such as testosterone, LH, and FSH, for the various groups. According to the findings, there were no significant variations between the Cis^-^/AO^+^400 group and the control group; while the amount of markers in the Cis^+^/AO^-^ group decreased significantly (*p*<0.05) as compared to the control group. When compared to the control group, the Cis^+^/AO^+^100 group had a lower level of biochemical hormones among the AO-administered groups (*p*<0.05). In comparison with the Cis+/AO^-^ group, the amount of testosterone, LH, and FSH hormones in this group increased. Chemical hormonal levels in the Cis^+^/AO^+^200 group were significantly higher than those in the Cis^+^/AO^+^100 and Cis^+^/AO^-^ groups (*p*<0.05). When compared to the control group, this group showed a significant decrease in testosterone and LH hormone levels. In comparison with the Cis^+^/AO^+^100, Cis^+^/AO^+^200, and Cis^+^/AO^-^ groups, the amounts of hormones in Cis^+^/AO^+^400 increased significantly (*p*<0.05). When compared to the control group, this group showed a significant reduction in the level of biomarkers. When compared to the Cis^+^/AO^+^200 group, the testosterone, FSH, and LH hormone levels were significantly higher (*p*<0.05).

#: *p*<0.05 *vs*. control group

*: *p*<0.05 *vs*. Cis^+^/AO^-^

$: *p*<0.05 *vs*. Cis^+^/AO^+^100

a: *p*<0.05 *vs*. Cis^+^/AO^+^200.

### Oxidative stress

As shown in Table 1, the Cis^+^/AO^-^ group showed a significant increment in MDA levels. According to results from the Cis^-^/AO^+^400 group, the MDA level did not change significantly in relation to the control group. Moreover, the increase in the MDA level was significant in the Cis^+^/AO^-^ group (*p*<0.05). There was a reduction in the Cis^+^/AO^+^100 compared to the Cis^+^/AO^-^ group (*p*<0.05); however, the increase in the AO dosage (Cis^+^/AO^+^200 and Cis^+^/AO^+^400) did not change the MDA value significantly when compared to the Cis^+^/AO^+^100 group.

**Table 1:** Testis homogenate MDA contents and SOD activities among different studied groups.

Groups	MDA (nmol/g. tissue)	SOD (µ/ml)
Control	49.4±0.8^a^	4.56±0.5^a^
Cis^-^/AO^+^400	53.66±1.1^ab^	4.5±0.5^a^
Cis^+^/AO^-^	87.53±1.0^c^	4.53±0.5^a^
Cis^+^/AO^+^100	66.93±2.8^b^	10.8±0.9^c^
Cis^+^/AO^+^200	61.66±7.8^ab^	7.7±0.5^b^
Cis^+^/AO^+^400	55.36±3.7^ab^	6.9±0.5^ab^

Lowercase letters show a significant difference (*p*<0.05) between groups in each column.

The SOD activity in the Cis^+^/AO^-^ group did not change in the testes tissue when compared to the control group. Also, the SOD activity of the Cis^-^/AO^+^400 and Cis^+^/AO^-^ groups did not change in comparison with the control group. The Cis^+^/AO^+^100 group had a significant increment compared to the control group (*p*<0.05), and there was a further rise in SOD activity in the Cis^+^/AO^+^200 group. On the other hand, the change in SOD activity in the testes tissue was not significant in the Cis^+^/AO^+^400 group when compared to Cis^+^/AO^+^200; while it was significant when compared to the control and Cis^+^/AO^-^ groups.

### Histopathological analysis

**Figure 4:**
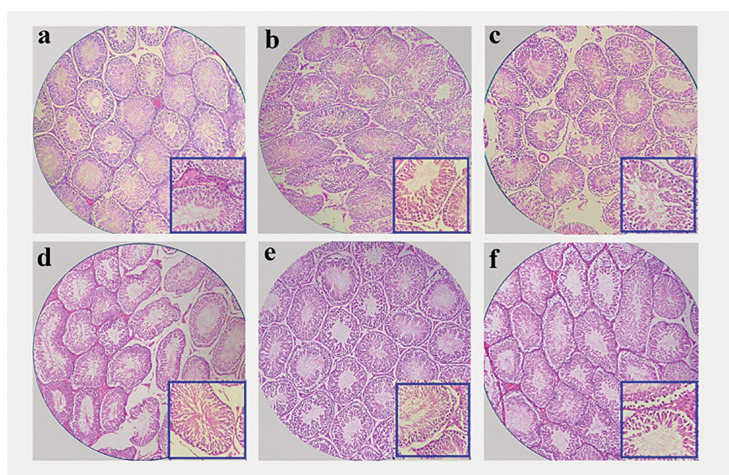
Testis tissue of the a) control, b) Cis^-^/AO+400, c) Cis^+^/AO^-^, d) Cis^-^/AO^+^100, e) Cis^-^/AO^+^200, and f) Cis^-^/AO^+^400 groups (magnification: ×100, inset: ×400).

Figure 4 depicts the testis tissue of the rats from the different groups. As shown, there no abnormalities in the control group testes, and a similar result was found in tissues related to the Cis^-^/AO^+^400 group. In these groups, the most differentiated germ cells were found in round spermatids. Histopathological sections of the Cis^+^/AO^-^ group showed degeneration, necrosis, and interstitial edema in testis tissue in comparison with the control group. Moreover, some seminiferous tubules underwent degeneration and were also noticeably depleted of germ cells relative to the control group. In addition, in the seminiferous epithelium there was accentuated cellular depletion. There was a reduction in germinal epithelium thickness. The changes in histopathologic findings caused by Cis administration were reversed by treatment with AO. The changes were not significant in the histopathologic structures among the groups administered Cis and AO.

## DISCUSSION

Changes in biochemical, histological, and molecular factors usually indicate male reproductive toxicity induced by AO (Narayana, 2008). To date, no successful antioxidant-mediated counteractive approach against the drugs’ side effects has been investigated. If we can create an antioxidant therapy regimen that can reduce the drugs’ side effects, it would be possible to use them more effectively in clinical practice.

The animals’ body weight reduction was caused by the medication toxicity, since they suffered from diarrhea and consumed limited water and food. A significant decrease in testis weight was expected as a result of the additive impact of the drugs; however, this was not the case. As a consequence, the Cis-based cumulative effects at clinical dosage levels are fewer than those seen in single-exposure trials with higher doses of individual drugs (Amin & Hamza, 2006). There was also testis weight reduction. It has been reported in previous studies that the testis weight was reduced along with anatomical alterations in both humans (Howell & Shalet, 2002) and animals after three cycles of human therapeutic dosage amounts of Cis alone, owing to the drug cytotoxicity (Sawhney *et al*., 2005).

AO was not able to inhibit or restore the weight loss, which may be attributed to an inability to regulate the processes that caused the extreme toxicity. Cytotoxicity mediated by oxidative stress resulted in a substantial reduction in testis weight. According to previous investigations, higher doses of Cis reduced testis weight abruptly in single injection trials (Ateşşahin *et al*., 2006), but the gradual and not too dramatic organ weight reduction occurred in this study, despite being important. This means that the strength of the drug doses determines the decrease in testis weight.

To assess male reproductive toxicity, specific testes hormones may be tested. Since the hormonal activities of the testes are 1000 times greater than those of the serum, the serum hormonal activity would be doubled if just 1% of male reproductive toxicity exists. The amounts of testosterone, LH, and FSH - which are biochemical markers of impaired testes tissue - were tested in this analysis to observe whether AO could help to avoid AO-induced male reproductive toxicity. The loss of functional integrity of the membrane of the male reproductive cells in reaction to harmful effects of certain treatments, as well as inflammatory disorders such as cirrhosis, leads to the leakage of these hormones, resulting in a reduction in hormonal levels. Accordingly, the amounts of biochemical hormonal marker in the Cis-administered group were the lowest. The levels of the hormones increased by the increase in the AO concentration revealing its effect on alleviating the adverse effects of Cis. Narayana *et al*. (2012) studied the early combined molecular influence of clinical doses of bleomycin, etoposide, and Cis (BEP) on the testis, as well as the effect of the AO antioxidant compound. Their findings revealed that molecular changes induced by BEP were recovered by AO to control levels. The activation of oxidative stress, induction of cell death, and up-regulation of proapoptotic proteins are all involved in BEP-induced early testicular injuries. AO greatly reduces the pathogenesis of testicular injury caused by BEP, implying that it can be used therapeutically.

The increase in MDA, as well as the reduction in testes antioxidants such as SOD activity, indicated testes injured induced by Cis. Previous studies have documented potential oxidative stress induction by Cis injection, which results in cell death in testes tissue. Overproduction of reactive oxygen species and/or lack of antioxidant mechanisms induce oxidative stress (Elchuri *et al*., 2005; Zhao *et al*., 2009). In normal metabolism, the testes remove formed reactive oxygen species by complex reactions involving enzymes such as catalase, SOD, peroxiredoxin, and GSH-Pxs. In testes cells exposed to oxidative stress, such as Cis-induced male reproductive toxicity, the produced amount of reactive oxygen species is more than the detoxification capacity. Protein oxidation (growth factor inhibition and reduced enzymatic functions), lipid peroxidation (radical inflammation and development), DNA damage (facilitated cell death and reduced proliferation) contribute to male reproductive toxicity damage (Li *et al*., 2011). Flavonoid (galangin) antioxidant compounds are responsible for the antioxidant impact of AO (Sani *et al*., 2019; Tungmunnithum *et al*., 2020). Free-radical scavenging activities of flavonoid lead to suppression of the chemical-mediated genotoxicity together with modulation of enzyme activities (Swain *et al*., 2020).

## CONCLUSION

To conclude, we showed the beneficial effect of AO administration on the reduction of Cis-induced male reproductive toxicity in rats. This is accomplished through a variety of intracellular pathways, including enhancing testes’ functional parameters, suppressing oxidative stress, and altering antioxidant protection mechanisms. Male reproductive damage was determined by a decrease in testosterone, LH, and FSH levels, which was significantly increased in AO-treated populations. In addition, AO therapy modified SOD and MDA levels. Overall, the Cis^+^/AO^+^400 group exhibited the best antioxidant properties. These findings could lead to changes in cancer care guidelines that incorporate phytochemicals, making cancer therapies safer.
